# Supplementary material for: Hybrid immunity improves B cells and antibodies against SARS-CoV-2 variants
Source: Nature. 2021 Oct 20;600(7889):530–5. doi: 10.1038/s41586-021-04117-7 (PMC8674140; doi:10.1038/s41586-021-04117-7)
Supplement: Supplementary file 1 — Reporting Summary [file 41586_2021_4117_MOESM1_ESM.pdf]

nature portfolio

Corresponding author(s): Rino Rappuoli

Last updated by author(s): Oct 3, 2021

Reporting Summary

Nature Portfolio wishes to improve the reproducibility of the work that we publish. This form provides structure for consistency and transparency in reporting. For further information on Nature Portfolio policies, see our Editorial Policies and the Editorial Policy Checklist.

Statistics

For all statistical analyses, confirm that the following items are present in the figure legend, table legend, main text, or Methods section.

n/a	Confirmed	
☐	☒	The exact sample size (*n*) for each experimental group/condition, given as a discrete number and unit of measurement
☐	☒	A statement on whether measurements were taken from distinct samples or whether the same sample was measured repeatedly
☐	☒	The statistical test(s) used AND whether they are one- or two-sided<br>*Only common tests should be described solely by name; describe more complex techniques in the Methods section.*
☒	☐	A description of all covariates tested
☒	☐	A description of any assumptions or corrections, such as tests of normality and adjustment for multiple comparisons
☐	☒	A full description of the statistical parameters including central tendency (e.g. means) or other basic estimates (e.g. regression coefficient) AND variation (e.g. standard deviation) or associated estimates of uncertainty (e.g. confidence intervals)
☐	☒	For null hypothesis testing, the test statistic (e.g. *F*, *t*, *r*) with confidence intervals, effect sizes, degrees of freedom and *P* value noted<br>*Give P values as exact values whenever suitable.*
☒	☐	For Bayesian analysis, information on the choice of priors and Markov chain Monte Carlo settings
☒	☐	For hierarchical and complex designs, identification of the appropriate level for tests and full reporting of outcomes
☒	☐	Estimates of effect sizes (e.g. Cohen's *d*, Pearson's *r*), indicating how they were calculated

Our web collection on statistics for biologists contains articles on many of the points above.

Software and code

Policy information about availability of computer code

Data collection

- Thermo Fisher SkanIt Software Microplate Readers 6.0.1
- BD Biosciences BD FACSDiva Software v9.0

Data analysis

- GraphPad Prism 8.0.2 was used to perform statistical analyses
- BD FlowJo 10.5.3
- Qiagen CLC sequence viewer 350 8.0.0
- Boston University, Cloanalyst (http://www.bu.edu/computationalimmunology/research/software/)

For manuscripts utilizing custom algorithms or software that are central to the research but not yet described in published literature, software must be made available to editors and reviewers. We strongly encourage code deposition in a community repository (e.g. GitHub). See the Nature Portfolio guidelines for submitting code & software for further information.

Data

Policy information about availability of data

All manuscripts must include a data availability statement. This statement should provide the following information, where applicable:

- Accession codes, unique identifiers, or web links for publicly available datasets
- A description of any restrictions on data availability
- For clinical datasets or third party data, please ensure that the statement adheres to our policy

Source data are provided with this paper. All data supporting the findings in this study are available within the article or can be obtained from the corresponding author upon request.

Field-specific reporting

Please select the one below that is the best fit for your research. If you are not sure, read the appropriate sections before making your selection.

☒ Life sciences ☐ Behavioural & social sciences ☐ Ecological, evolutionary & environmental sciences

For a reference copy of the document with all sections, see nature.com/documents/nr-reporting-summary-flat.pdf

Life sciences study design

All studies must disclose on these points even when the disclosure is negative.

Sample size	10 subjects in total, 5 seronegative and 5 seropositive, were analyzed in this study. A total of 2,352 and 3,532 spike protein specific memory B cells from seronegative and seropositive subjects were tested in this study. Given the exploratory nature of the study, we did not use statistical methods to predetermine sample size. Sample size was based on previous studies that applied a similar technology. The authors believed that 5 subjects/group were a good balance between feasibility of analyzing at single cell level several thousands of memory B cells and the ability to represent the antibody response of seronegative and seropositve people.
Data exclusions	No data was excluded.
Replication	All experiments were performed in technical duplicates or triplicates as indicated in the figure legends and methods section.
Randomization	The experiments were not randomized and all available samples were tested. The authors aimed to specifically assess the antibody response of seronegative and seropositve subjects. Donors were specifically recruited based on their previous infection and vaccination history. Randomization would have not allowed to enroll 5 subjects/group which was our technical limit for single cell analysis of the antibody response. Based on what stated above, the authors believed that randomization was not appropriate.
Blinding	The investigators were not blinded during group allocation, data collection and analyses. The clinical protocol established to enroll subjects in this study reports information regarding previous infection and vaccination in order to allocate 5 subjects/group. Pseudonymized information received in the lab reports the same information and therefore blinding for group allocation was not possible.

Reporting for specific materials, systems and methods

We require information from authors about some types of materials, experimental systems and methods used in many studies. Here, indicate whether each material, system or method listed is relevant to your study. If you are not sure if a list item applies to your research, read the appropriate section before selecting a response.

Materials & experimental systems

n/a	Involved in the study	
☐	☒	Antibodies
☐	☒	Eukaryotic cell lines
☒	☐	Palaeontology and archaeology
☒	☐	Animals and other organisms
☐	☒	Human research participants
☒	☐	Clinical data
☒	☐	Dual use research of concern

Methods

n/a	Involved in the study	
☒	☐	ChIP-seq
☐	☒	Flow cytometry
☒	☐	MRI-based neuroimaging

Antibodies

Antibodies used	BD Biosciences CD19 BV421, Cat#562440, Clone ID HIB19, Lot#8270584<br>BD Biosciences IgM PerCP-Cy5.5, Cat#561285, Clone ID G20-127, Lot#9269055<br>BD Biosciences CD27 PE, Cat#340425, Clone ID L128, Lot#9288842<br>BD Biosciences IgD-A700, Cat#561302, Clone ID IA6-2, Lot#9199226<br>BioLegend CD3 PE-Cy7, Cat#300420, Clone ID UCHT1, Lot#B303315<br>BioLegend CD14 PE-Cy7, Cat#301814, Clone ID M5E2, Lot#B272337<br>BioLegend CD56 PE-Cy7, Cat#318318, Clone ID HCD56, Lot#B297987<br>Southern Biotech Goat Anti-Human IgG-Alkaline Phosphatase, Cat#2040-04, polyclonal, Lot#K2119-XG00B<br>Southern Biotech Goat Anti-Human IgA-Alkaline Phosphatase, Cat#2050-04, polyclonal, Lot#G0919-W620C<br>Sigma-Aldrich Anti-Human IgG (Fab specific)–Peroxidase antibody produced in goat, Cat#A0293, polyclonal, Lot#019M4876V
Validation	BD Biosciences CD19 BV421, Cat#562440, Clone ID HIB19, QC testing, reactivity human, application flow cytometry (https://www.bdbiosciences.com/content/bdb/paths/generate-tds-document.us.562440.pdf).<br>BD Biosciences IgM PerCP-Cy5.5, Cat#561285, Clone ID G20-127, QC testing, reactivity human, application flow cytometry (https://www.bdbiosciences.com/content/bdb/paths/generate-tds-document.us.561285.pdf).<br>BD Biosciences CD27 PE, Cat#340425, Clone ID L128, QC testing, reactivity human, application flow cytometry (https://

www.bdbiosciences.com/en-us/products/reagents/flow-cytometry-reagents/clinical-discovery-research/single-color-antibodies-ruo-gmp/pe-mouse-anti-human-cd27.340425)
BD Biosciences IgD-A700, Cat#561302, Clone ID IA6-2, QC testing, reactivity human, application flow cytometry (https://www.bdbiosciences.com/content/bdb/paths/generate-tds-document.us.561302.pdf)
BioLegend CD3 PE-Cy7, Cat#300420, Clone ID UCHT1, reactivity human and cross-reactivity with chimpanzee, application flow cytometry (https://www.biolegend.com/en-us/global-elements/pdf-popup/pe-cyanine7-anti-human-cd3-antibody-3070?filename=PECyanine7%20anti-human%20CD3%20Antibody.pdf&pdfgen=true)
BioLegend CD14 PE-Cy7, Cat#301814, Clone ID M5E2, Reactivity Human, African Green, Capuchin Monkey, Cattle (Bovine, Cow), Chimpanzee, Common Marmoset, Cotton-topped Tamarin, Cynomolgus, Dog (Canine), Rhesus, Pigtailed Macaque, Squirrel Monkey, application flow cytometry (https://www.biolegend.com/en-us/global-elements/pdf-popup/pe-cyanine7-anti-human-cd14-antibody-2729?filename=PECyanine7%20anti-human%20CD14%20Antibody.pdf&pdfgen=true)
BioLegend CD56 PE-Cy7, Cat#318318, Clone ID HCD56, Reactivity Human, African Green, Baboon, Cynomolgus, Rhesus, application flow cytometry (https://www.biolegend.com/en-us/global-elements/pdf-popup/pe-cyanine7-anti-human-cd56-ncam-antibody-3802?filename=PECyanine7%20anti-human%20CD56%20NCAM%20Antibody.pdf&pdfgen=true)
Southern Biotech Goat Anti-Human IgG-Alkaline Phosphatase, Cat#2040-04, polyclonal, reactivity heavy chain of human IgG, application ELISA (https://www.southernbiotech.com/techbul/2040.pdf)
Southern Biotech Goat Anti-Human IgA-Alkaline Phosphatase, Cat#2050-04, polyclonal, reactivity heavy chain of human IgA, application ELISA (https://www.southernbiotech.com/techbul/2050.pdf)
Sigma-Aldrich Anti-Human IgG (Fab specific)–Peroxidase antibody produced in goat, Cat#A0293, polyclonal, reactivity human, application ELISA (https://www.sigmaaldrich.com/IT/en/product/sigma/a0293#)

Eukaryotic cell lines

Policy information about cell lines

Cell line source(s)

VERO E6 cell line ATCC Cat#CRL-1586; Expi293F cells Thermo Fisher Cat#A14527; 3T3-msCD40L Cells NIH AIDS Reagent Program Cat#12535.

Authentication

These cell lines were obtained from vendors that sell authenticated cell lines, they grew, performed and showed morphology as expected. No additional specific authentication was performed.

Mycoplasma contamination

Vero E6 cell lines are routinely tested on a monthly basis and tested negative for mycoplasma. 3T3-msCD40L cell line was tested negative to mycoplasma by the provider and Expi293F cells were not tested for mycoplasma contamination.

Commonly misidentified lines
(See ICLAC register)

No commonly misidentified cell lines were used in this study.

Human research participants

Policy information about studies involving human research participants

Population characteristics

This work results from a collaboration with the Azienda Ospedaliera Universitaria Senese, Siena (IT) that provided samples from COVID-19 vaccinated donors, of both sexes (4 females and 6 males), who gave their written consent. All data relevant to enrolled subjects are reported in this study. Subjects elegible for this study were of all sexes (aged 18-85) naïve or previously infected by SARS-CoV-2 and then vaccinated with the COVID-19 BNT162b2 mRNA vaccine.

Recruitment

Individuals with or without previous SARS-CoV-2 infection vaccinated with the COVID-19 BNT162b2 mRNA vaccine were enrolled by the clinicians involved in the study entitled "Isolamento di anticorpi monoclonali umani contro SARS-CoV-2 per lo sviluppo di nuove terapie e vaccini", Prot. n. TLS_SARS-CoV-2, at the Azienda Ospedaliera Universitaria Senese, Siena (IT). The authors do not see any potential bias in the generation or interpretation of the data reported in this study.

Ethics oversight

The study was approved by the Comitato Etico di Area Vasta Sud Est (CEAVSE) ethics committees (Parere 17065 in Siena) and conducted according to good clinical practice in accordance with the declaration of Helsinki (European Council 2001, US Code of Federal Regulations, ICH 1997). This study was unblinded and not randomized. No statistical methods were used to predetermine sample size.

Note that full information on the approval of the study protocol must also be provided in the manuscript.

Flow Cytometry

Plots

Confirm that:

☒ The axis labels state the marker and fluorochrome used (e.g. CD4-FITC).

☒ The axis scales are clearly visible. Include numbers along axes only for bottom left plot of group (a 'group' is an analysis of identical markers).

☒ All plots are contour plots with outliers or pseudocolor plots.

☒ A numerical value for number of cells or percentage (with statistics) is provided.

Methodology

Sample preparation

Human PBMC were isolated from heparin-treated whole blood by density gradient centrifugation (Ficoll-Paque™ PREMIUM,

Sample preparation	Sigma-Aldrich). After separation, PBMC were stained with Live/Dead Fixable Aqua (Invitrogen; Thermo Scientific) diluted 1:500 at room temperature RT. After 20 min incubation cells were washed with PBS and unspecific bindings were saturated with 20% normal rabbit serum (Life technologies). Following 20 min incubation at 4°C cells were washed with PBS and stained with SARS-CoV-2 S-protein labeled with Strep-Tactin®XT DY-488 (iba-lifesciences cat# 2-1562-050) for 30 min at 4°C. After incubation the following staining mix was used CD19 V421 (BD cat# 562440, 1:320), IgM PerCP-Cy5.5 (BD cat# 561285, 1:50), CD27 PE (BD cat# 340425, 1:30), IgD-A700 (BD cat# 561302, 1:15), CD3 PE-Cy7 (BioLegend cat# 300420, 1:100), CD14 PE-Cy7 (BioLegend cat# 301814, 1:320), CD56 PE-Cy7 (BioLegend cat# 318318, 1:80) and cells were incubated at 4°C for additional 30 min. Stained MBCs were single cell-sorted with a BD FACS Aria III (BD Biosciences).
Instrument	BD FACS Aria III Cell Sorter BD Biosciences
Software	BD Biosciences BD FACSDiva Software v9.0
Cell population abundance	Single cell sorted S protein trimer-specific (S protein+), class-switched memory B cells (CD19+CD27+IgD-IgM-) were 0.21, 0.25, 0.35, 0.39, 0.20, 0.57, 0.62, 0.67, 0.40 and 1.19% for subject VAC-001, VAC-002, VAC-007, VAC-008, VAC-010, VAC-003, VAC-004, VAC-005, VAC-006 and VAC-009 respectively. Sorted cells were gated on the CD19+CD27+IgD-IgM-S protein+ population based on the negative control as reported in the Extended Data (Extended Data Figure 1a,b).
Gating strategy	The gating strategy used for the single cell sorting of spike protein specific memory B cells is shown in the Extended Data (Extended Data Figure 1). Boundaries between "positive" and "negative" cells are defined and denoted on each graph.

☒ Tick this box to confirm that a figure exemplifying the gating strategy is provided in the Supplementary Information.
